# Identification of a Prognostic 3-Gene Risk Prediction Model for Thyroid Cancer

**DOI:** 10.3389/fendo.2020.00510

**Published:** 2020-08-06

**Authors:** Haiping Zhao, Shiwei Zhang, Shijie Shao, Haixing Fang

**Affiliations:** Department of General Surgery, The First People's Hospital of Fuyang, Hangzhou, China

**Keywords:** THCA, WGCNA, prognostic 3-gene risk prediction model, prediction, prognosis

## Abstract

**Objective:** We aimed to screen the genes associated with thyroid cancer (THCA) prognosis, and construct a poly-gene risk prediction model for prognosis prediction and improvement.

**Methods:** The HTSeq-Counts data of THCA were accessed from TCGA database, including 505 cancer samples and 57 normal tissue samples. “edgeR” package was utilized to perform differential analysis, and weighted gene co-expression network analysis (WGCNA) was applied to screen the differential co-expression genes associated with THCA tissue types. Univariant Cox regression analysis was further used for the selection of survival-related genes. Then, LASSO regression model was constructed to analyze the genes, and an optimal prognostic model was developed as well as evaluated by Kaplan-Meier and ROC curves.

**Results:** Three thousand two hundred seven differentially expressed genes (DEGs) were obtained by differential analysis and 23 co-expression genes (|COR| > 0.5, *P* < 0.05) were gained after WGCNA analysis. In addition, eight genes significantly related to THCA survival were screened by univariant Cox regression analysis, and an optimal prognostic 3-gene risk prediction model was constructed after genes were analyzed by the LASSO regression model. Based on this model, patients were grouped into the high-risk group and low-risk group. Kaplan-Meier curve showed that patients in the low-risk group had much better survival than those in the high-risk group. Moreover, great accuracy of the 3-gene model was revealed by ROC curve and the remarkable correlation between the model and patients' prognosis was verified using the multivariant Cox regression analysis.

**Conclusion:** The prognostic 3-gene model composed by *GHR, GPR125*, and *ATP2C2* three genes can be used as an independent prognostic factor and has better prediction for the survival of THCA patients.

## Introduction

Thyroid cancer (THCA), derived from parafollicular cells or thyroid follicular cells, is the most common endocrine malignancy accounting for about 1% of all kinds of human cancers (1). Papillary (PTC), follicular, anaplastic and medullary thyroid carcinomas are the four subtypes of THCA (2), among which papillary and follicular carcinomas are common and have better prognosis (3), while anaplastic carcinoma is rare to be seen with extremely poor prognosis (4). Therefore, it's very important to find effective approaches for the improvement of the overall THCA prognosis.

At present, the conventional prognostic model of THCA in clinical practice is constructed according to predictive factors like age, tumor size and lymph nodule metastasis (5). With the development of high-throughput sequencing technology, mRNA expression profiles of specific cancers are easy to obtain, which helps us better find more robust prognostic signals (6). For instance, microarray-based gene expression analysis enables us to identify the important genes during tumor progression and helps to define and diagnose prognostic characteristics (7). In this way, many THCA prognostic biomarkers have been verified. However, these markers are almost single genes and have not been widely accepted (8). Polygenic combination has been reported to possess better predictive ability for cancer prognosis than single genes (9). Therefore, recent studies have involved in the identification of the biomarkers for THCA prognosis (10). However, restricted by research methods, novel biological algorithm needs to be explored to construct more accurate diagnostic or prognosis models.

In the present study, a large number of mRNA expression profiles of THCA patients were accessed from TCGA database, and modules associated with THCA were identified by WGCNA. A 3-gene risk prediction model was constructed using Cox and LASSO regression models, which could help us better predict THCA prognosis.

## Materials and Methods

### Data Resource

Expression profiles of THCA mRNA and corresponding clinical data were accessed from TCGA database (https://cancergenome.nih.gov/), including 506 cancer samples and 56 normal tissue samples. The study was in line with the guidelines released by TCGA (http://cancergenome.nih.gov/publications/publicationguidelines).

### Identification and Confirmation of THCA-Associated Genes

“edgeR” package (https://bioconductor.org/packages/release/bioc/html/edgeR.html) was used to perform differential analysis between cancer tissues and normal tissues. Genes met the criteria (|logFC| > 1 and *P* < 0.05) were considered to have significant differences.

### Module Selection With WGCNA

The mechanism of WGCNA is the research for co-expression modules and the exploration of the correlation between the gene network and the phenotypes, which is motivated by the analyses of scale-free clustering and dynamic tree cut on expression profiles. In the present study, modules that were most related to THCA tissue types in the co-expression network constructed by WGCNA package (https://cran.r-project.org/web/packages/WGCNA/index.html) were selected, and genes meeting *P* < 0.05 and |COR| > 0.5 were extracted for further study.

### Construction of the Prognostic Risk Prediction Model

THCA prognosis-associated genes were screened using univariant Cox regression analysis. Then, a prognostic model was constructed using the least absolute shrinkage and selection operator (LASSO). According to this model, risk score of each sample was calculated, and patients were divided into the high-risk group and low-risk group with the median risk score as the threshold. Kaplan-Meier was used to evaluate the survival of the two groups. The ROC curve was drawn for the evaluation of the prognosis performance of the model, and the area under the curve (AUC) was calculated. Furthermore, multivariant Cox regression analysis was performed to assess the correlation between the risk score and patients' prognosis. Kaplan-Meier and ROC curves of each gene in this model were plotted to make a comparison with those curves of the model.

### Statistical Analysis

Univariant and multivariant Cox regression analyses were both performed in TCGA dataset. “glmnet” package of the R software (https://www.r-project.org/) was used for LASSO statistic algorithm. IBM SPSS 22.0 statistical software (IBM Corp., Armonk, NY, USA) was applied for statistical analysis. *P* < 0.05 was considered statistically significant.

## Results

### Identification of THCA-Associated Modules

**Figure 1:**
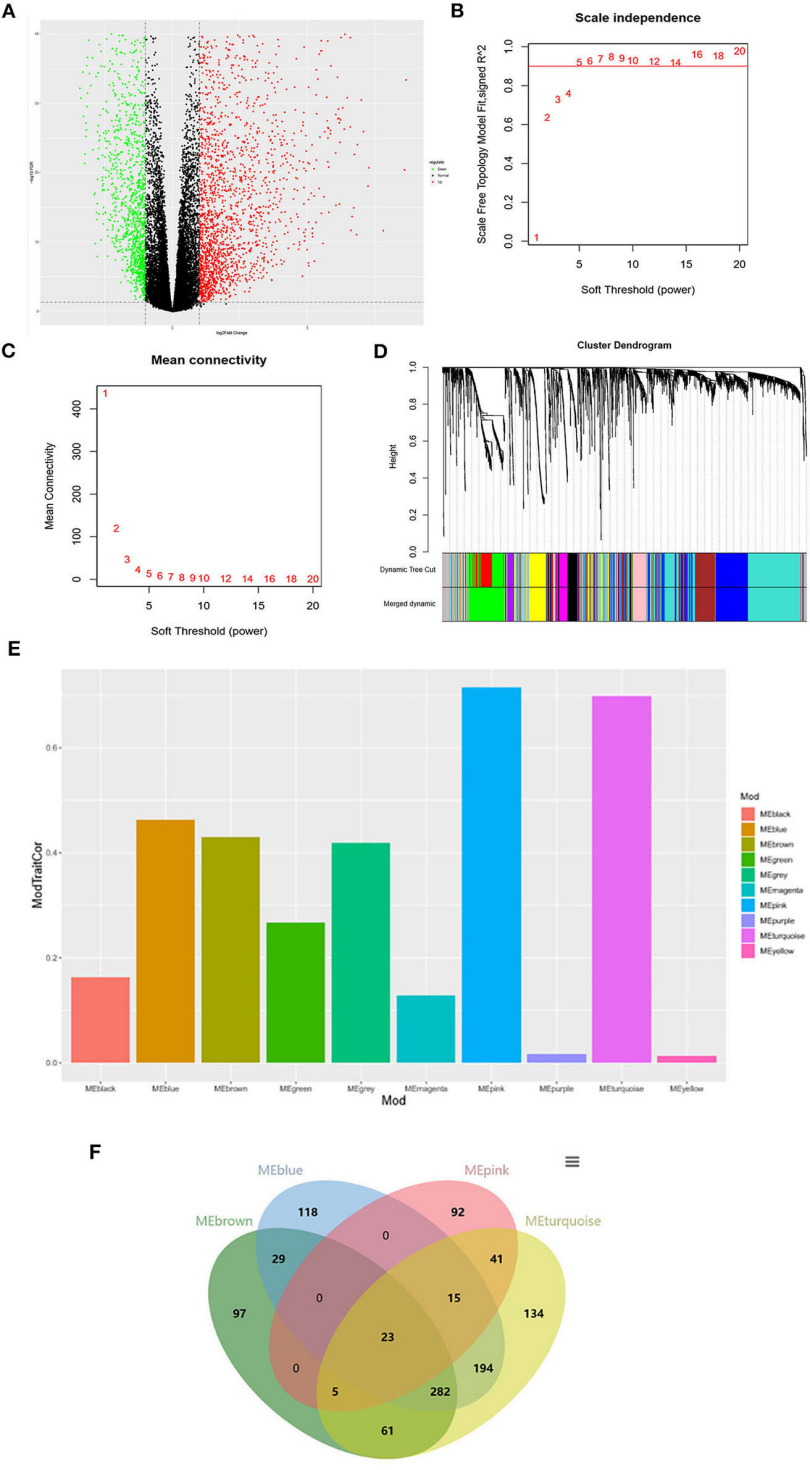
Identification of the THCA tissue type-associated RNA functional modules. **(A)** Volcano plot of DEGs; **(B)** Analysis of scale-independence index for various soft threshold powers. Horizontal axis is the soft threshold (power), and vertical axis is the scale-free topology fitting indices (*R*^2^). The red line refers to the standard corresponding to the *R*^2^ of 0.9; **(C)** Analysis of the mean connectivity under different soft threshold powers; **(D)** Cluster dendrogram of all DEGs clustered based on a dissimilarity measure; **(E)** Distribution of average gene significance and errors in the modules associated with the progression of THCA; **(F)** Venn diagram of the genes in the four modules for co-expression genes selection.

As shown in Figure 1A, a total of 3207 DEGs were identified (|logFC| > 1, *P* < 0.05). WGCNA was used to screen THCA related modules, and appropriate adjacency matrix weight parameter β (power) was selected to ensure the scale-free distribution of the co-expression network as possible (11). In the range of 1 ≤ β ≤ 20, log k and log P(k) were calculated for linear models' construction, respectively. β is the squared value of the coefficient R. As shown in Figure 1B, the soft threshold (power) is higher with the elevated *R*^2^, suggesting that the network closely approaches to scale-free distribution. In the present study, β = 5 (*R*^2^ = 0.9 for the first time) was selected to ensure the realization of scale-free distribution as possible and make the values on the curve approach to the minimum threshold. When β = 5, the mean connectivity of RNA in the network was 5 (Figure 1C), which was consistent with the small-world network in the scale-free one. Then, cluster dendrogram was constructed (Figure 1D) and dynamic tree cut was performed (deep split = 2). Modules obtained were merged with the minimum size of 50, and 10 modules were eventually developed.

The correlation and significance between the module characteristics and sample phenotypes were calculated. Among the 10 modules, genes in blue, brown, pink and turquoise modules were verified to be most associated with THCA prognosis (Figure 1E). 23 THCA tissue type-associated genes were obtained from the four modules taking the *P* < 0.05 and |COR| > 0.5 as the threshold (Figure 1F).

### Construction of a Prognostic 3-Gene Risk Prediction Model for THCA

**Figure 2:**
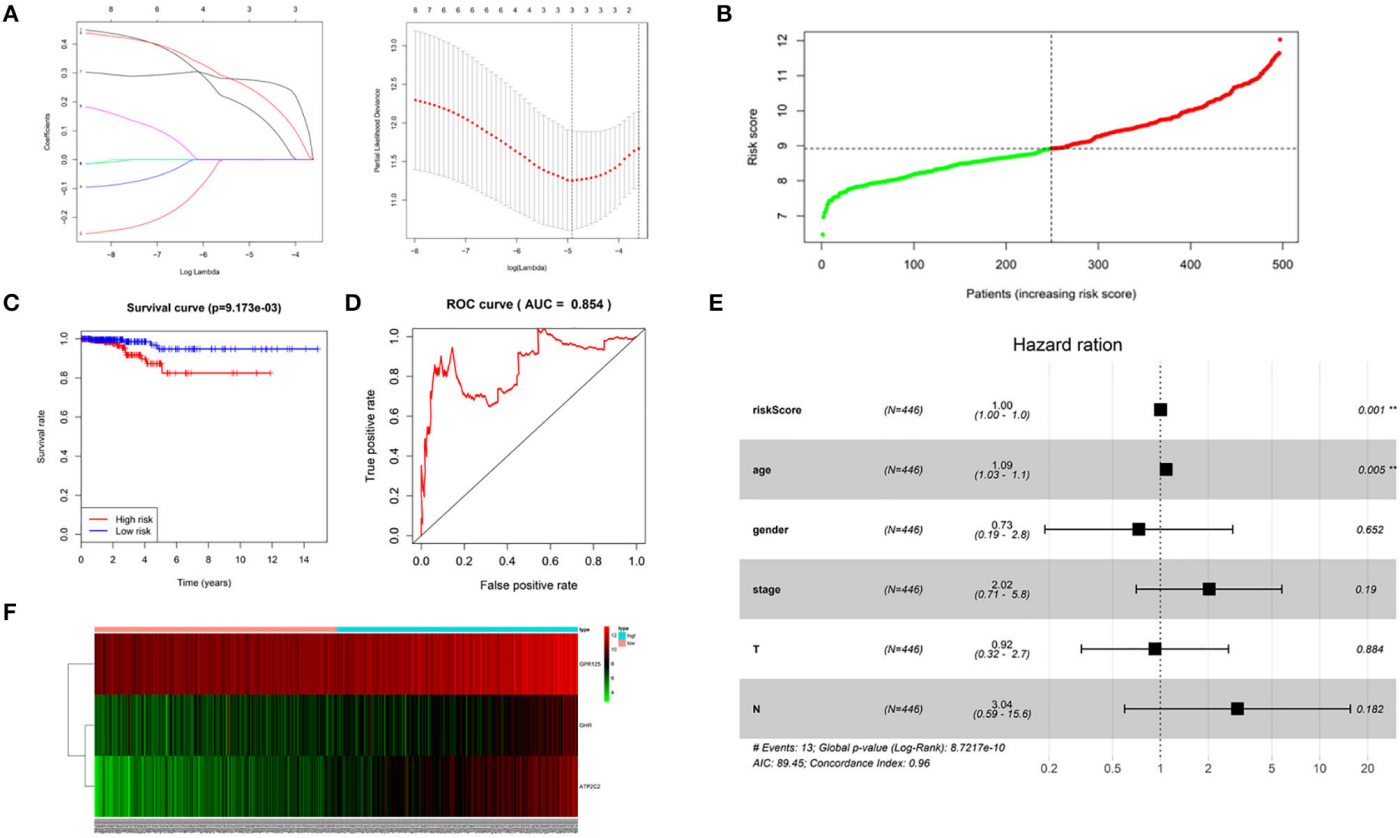
Construction of a 3-gene risk prediction model. **(A)** LASSO regression model; **(B)** 3-gene based distribution of risk scores; **(C)** Survival analysis of real hub genes in the TCGA-THCA dataset; **(D)** ROC curve of real hub genes in the TCGA-THCA dataset; **(E)** The correlation between the risk score and patients' prognosis; **(F)** Heatmap of the 3 genes expression profiles.

Univariant Cox regression was performed for analysis of the 23 co-expression genes, suggesting that eight genes were significantly correlated with survival as shown in Table 1. LASSO regression model was constructed to analyze the genes and an optimal prognostic risk prediction model was eventually developed (Figure 2A). Risk Score = (0.185780133850552 × GHR) + (0.277546742101366 × GPR125) + (0.257150281664915 × Atp2c2). Risk prediction was performed according to this model, and patients were ranged based on the risk scores (Figure 2B). The median risk score was used as the critical value to group the patients into the high-risk group (*n* = 248) and low-risk group (*n* = 249). As shown in the Kaplan-Meier curve in Figure 2C, patients in the high-risk group had worse overall survival (OS) than those in the low-risk group. ROC curve was plotted to predict the 3-year survival and the results showed in Figure 2D revealed that AUC of the 3-gene model was 0.854, which indicated the good performance of the risk score in survival prediction. Multivariant Cox proportional hazards regression analysis was then performed combined with clinical factors and the correlation between the risk score and prognosis of patients was verified (Figure 2E). From the heat maps of the expression profiles of these three genes (Figure 2F), the expression levels of *GHR, GPR125*, and *Atp2c2* were found to be positively correlated with the risk score, and all of them were regarded as high-risk genes.

**Table 1:** Basic information of the eight prognostic genes.

**id**	**HR**	**HR.95L**	**HR.95H**	***P*-value**
Atp2c2	1.767169	1.266988	2.464812	0.000797
GPR125	2.544272	1.465675	4.416615	0.000905
GHR	2.11237	1.316811	3.38857	0.001927
CLMN	1.794182	1.11235	2.893954	0.016554
CYTH3	2.402596	1.039985	5.550527	0.040195
PLA2R1	1.426271	1.008296	2.01751	0.044786
RYR2	1.278786	1.003806	1.629094	0.046512
C8orf88	1.551333	1.002698	2.400158	0.048602

### Evaluation of the 3-Gene Risk Prediction Model

**Figure 3:**
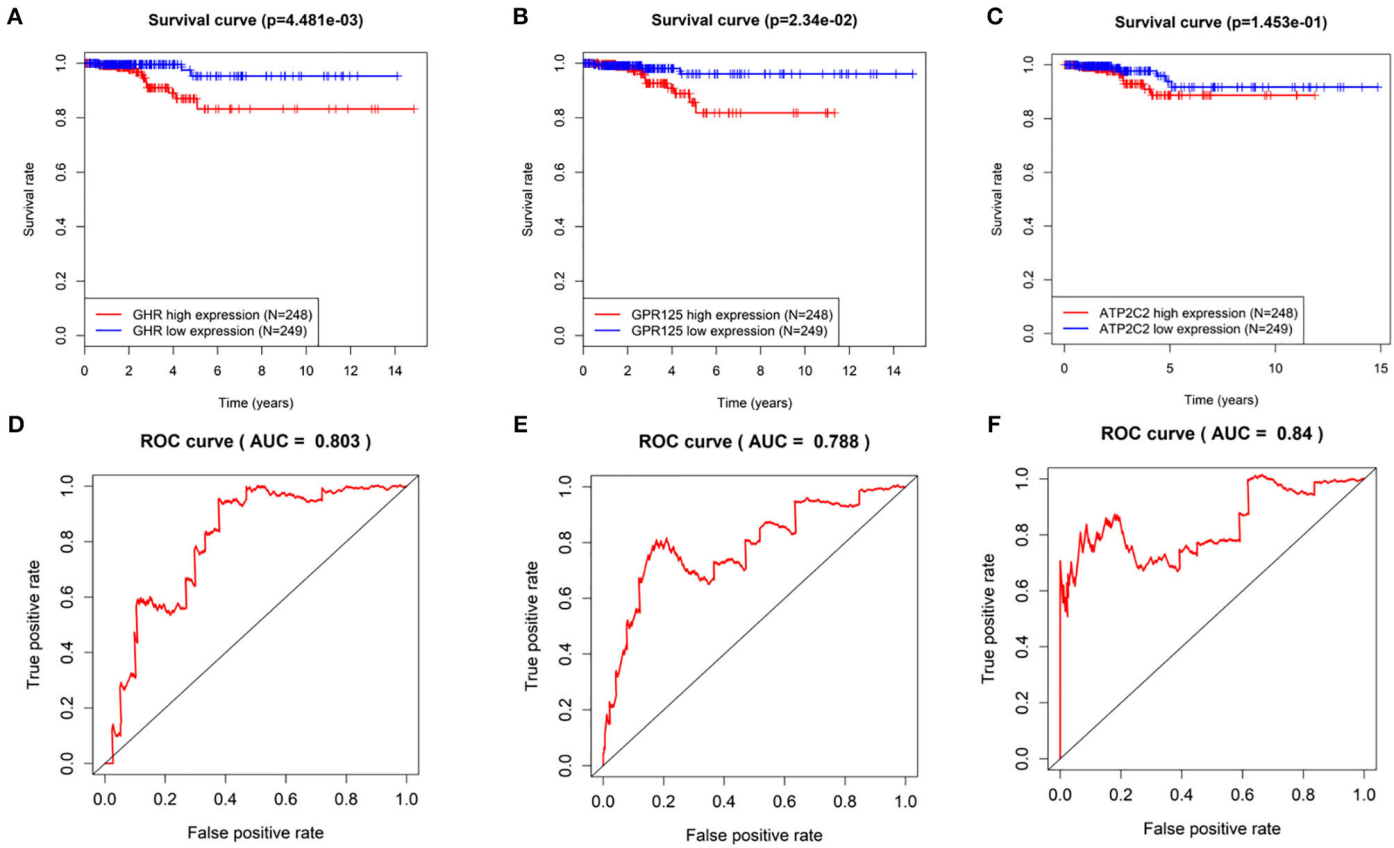
The evaluation of the 3-gene risk prediction model. **(A–C)** Survival analyses of GHR, GPR125, and Atp2c2 in the TCGA-THCA dataset; **(D–F)** ROC analyses of GHR, GPR125 and Atp2c2 in the TCGA-THCA dataset.

Kaplan-Meier curves of the three genes were drawn using the log rank test. As shown in Figures 3A–C, THCA patients with low expression of *GHR, GPR125*, and *Atp2c2* had longer survival time, indicting that these three genes were high-risk genes, which was in agreement with the results predicted by univariant Cox regression analysis. Furthermore, ROC curves (Figures 3D–F) revealed that the AUC of *GHR, GPR125*, and *Atp2c2* was 0.803, 0.788, and 0.84, respectively, all of which were smaller than that of the 3-gene risk prediction model. Findings above demonstrate that risk score is a good indicator for prognosis, and the 3-gene model has a higher accuracy.

## Discussion

With the development of the microarray and RNA sequencing technologies, new era of large data on biology is coming. It has been reported that microarray-based gene expression analysis could achieve characterization in human cancers, identification of the important genes during tumorigenesis and the definition as well as the diagnosis of prognostic features (7). However, the role of genes as prognosis factors has been few investigated (12). In the present study, a large amount of RNA-seq profiles and clinical prognosis data of THCA patients were accessed from TCGA database, and co-expression gene modules were screened using WGCNA. Studies have shown that gene modules are much reliable in cancer prognosis than biomarkers. While there are few studies on the cross-talk among the modules, and some important modules might be ignored (13). Therefore, in our study, gene co-expression network was constructed via WGCNA, and was used to identify THCA tissue type-associated gene modules, including blue, brown, pink and turquoise. Twenty-three common genes were obtained from the four modules, and an optimal prognostic 3-gene risk prediction model was then constructed by univariant Cox and LASSO regression analyses. Along with the LASSO model, all independent variables can be processed simultaneously, verifying the more accurate performance than the stepwise regression model (14). *GHR, GPR125*, and *Atp2C2* were the three genes in this model. *GHR* is a kind of protein-coding gene coding transmembrane receptors of the growth hormone. In prior studies, *GHR* has been verified to be a oncogene in some cancers, such as breast cancer (15), pancreatic ductal carcinoma (16) and melanoma (17), but the role in THCA prognosis is firstly reported. *GPR125*, a 57-KDa factor for transmembrane signal transduction, is considered to play a key role in cell adhesion and signal transduction (18). It's reported that *GPR125* is up-regulated in human cerebral cancer tissues (19) and promotes cell adhesion as well as the formation of myelosarcoma (20). In our study, *GHR* and *GPR125* were verified as high-risk genes in THCA, which was consistent with the previous studies. Moreover, we found that these two genes could be used as independent risk predictive factors, but the accuracy was lower than that of the 3-gene risk prediction model, which was further verified by ROC and Kaplan-Meier curves.

As the expression profiles of THCA and clinical information are just from one dataset of TCGA, the samples for analyzing the prognostic 3-gene model are limited. In addition, the model constructed in this study might be not available when it comes to other databases, and it's necessary to improve the model with more datasets. In a word, a 3-gene model is constructed to be an independent predictor in this study, which provides novel view and approach for the prognosis of THCA patients.

## Data Availability Statement

All datasets generated for this study are included in the article/supplementary material.

## Author Contributions

HZ contributed to the study design and gave the final approval of the version to be submitted. SZ conducted the literature search and performed data analysis and drafted. SS acquired the data and revised the article. HF wrote the article. All authors contributed to the article and approved the submitted version.

## Conflict of Interest

The authors declare that the research was conducted in the absence of any commercial or financial relationships that could be construed as a potential conflict of interest.
